# Retraction statement: MicroRNA‐21 promotes glioma cell proliferation and inhibits senescence and apoptosis by targeting SPRY1 via the PTEN/PI3K/AKT signaling pathway

**DOI:** 10.1111/cns.13614

**Published:** 2021-01-25

Retraction: Chai, C, Song, L‐J, Han, S‐Y, Li, X‐Q, Li, M. MicroRNA‐21 promotes glioma cell proliferation and inhibits senescence and apoptosis by targeting SPRY1 via the PTEN/PI3K/AKT signaling pathway. *CNS Neurosci Ther*. 2018; 24: 369–380. https://doi.org/10.1111/cns.12785

The above article, published online on January 05, 2018, in Wiley Online Library (wileyonlinelibrary.com), has been retracted by agreement between Shuangyin Han and Ming Li, the journal Editor‐in‐Chief Jun Chen, and John Wiley & Sons Ltd. The retraction has been agreed upon because the article was submitted and approved for publication by Chang Chai without consent in any form by the named co‐authors Shuangyin Han and Ming Li.

